# Loneliness: An Immunometabolic Syndrome

**DOI:** 10.3390/ijerph182212162

**Published:** 2021-11-19

**Authors:** Homa Pourriyahi, Niloufar Yazdanpanah, Amene Saghazadeh, Nima Rezaei

**Affiliations:** 1 Student Research Committee, School of Medicine, Iran University of Medical Sciences, Tehran 14496 14535, Iran; homapou99@gmail.com; 2 Systematic Review and Meta-Analysis Expert Group (SRMEG), Universal Scientific Education and Research Network (USERN), Tehran 14197 33151, Iran; 3 School of Medicine, Tehran University of Medical Sciences, Tehran 14167 53955, Iran; yazdanpanah.niloufar@yahoo.com; 4 Network of Immunity in Infection, Malignancy and Autoimmunity (NIIMA), Universal Scientific Education and Research Network (USERN), Tehran 14197 33151, Iran; 5 Research Center for Immunodeficiencies, Children’s Medical Center, Tehran University of Medical Sciences, Tehran 14197 33151, Iran; amene.saghazadeh@gmail.com; 6 MetaCognition Interest Group (MCIG), Universal Scientific Education and Research Network (USERN), Tehran 14197 33151, Iran; 7 Department of Immunology, School of Medicine, Tehran University of Medical Sciences, Tehran 14167 53955, Iran

**Keywords:** loneliness, perceived social isolation, psychosocial stress, CTRA, immune regulation, cytokines, antiviral immunity, metabolic regulation, stress circuitry, hypothalamic-pituitary-adrenocortical axis, cortisol

## Abstract

Loneliness has been defined as an agonizing encounter, experienced when the need for human intimacy is not met adequately, or when a person’s social network does not match their preference, either in number or attributes. This definition helps us realize that the cause of loneliness is not merely being alone, but rather not being in the company we desire. With loneliness being introduced as a measurable, distinct psychological experience, it has been found to be associated with poor health behaviors, heightened stress response, and inadequate physiological repairing activity. With these three major pathways of pathogenesis, loneliness can do much harm; as it impacts both immune and metabolic regulation, altering the levels of inflammatory cytokines, growth factors, acute-phase reactants, chemokines, immunoglobulins, antibody response against viruses and vaccines, and immune cell activity; and affecting stress circuitry, glycemic control, lipid metabolism, body composition, metabolic syndrome, cardiovascular function, cognitive function and mental health, respectively. Taken together, there are too many immunologic and metabolic manifestations associated with the construct of loneliness, and with previous literature showcasing loneliness as a distinct psychological experience and a health determinant, we propose that loneliness, in and of itself, is not just a psychosocial phenomenon. It is also an all-encompassing complex of systemic alterations that occur with it, expanding it into a syndrome of events, linked through a shared network of immunometabolic pathology. This review aims to portray a detailed picture of loneliness as an “immunometabolic syndrome”, with its multifaceted pathology.

## 1. Introduction

As social creatures, our need for social interaction, social support, and social stimuli should be met adequately in order for us to feel well and live a healthy life [1,2,3,4]. However, the objective presence of these social constructs does not always equal to the feeling of belonging; this feeling of belonging or social integration, rather comes with the psychological impact and individual perception of social relations and interactions [5], and if for any reason the individual does not perceive themselves as one who belongs, the feeling of loneliness arises [6].

Loneliness has been defined as an agonizing encounter, experienced when the need for human intimacy is not met adequately [7], or when a person’s social network doesn’t match their preference, either in number or attributes [8]. This definition helps us realize that the cause of loneliness isn’t merely being alone, but rather not being in the company we desire; meaning the feeling of loneliness and the state of social isolation are inherently different phenomena [9]. In other words, loneliness only occurs with the *perception of being lonely*, in spite of interpersonal relations, or a lack thereof [10].

As a subjective experience, loneliness can reflect the differences in patterns of thought, behavior, and situational reactions between people. These differences are under the influence of one’s inherent characteristics or dispositional traits and their individual adaptations to their experienced environments, i.e., the construct known as personality [11]. With the fundamental influence of personality on the emergence of the feeling of loneliness, the individual and cultural differences in coping mechanisms and in what is considered to be a satisfactory social relational status, together, play a dominant role in predicting loneliness and finding ways to mitigate it when designing or implementing intervention strategies [12].

Although there is research that does not believe loneliness to be a distinct phenomenon of its own, describing it as part of an indistinguishable interrelated spectrum of feelings [13], the development of a scale for measurement of loneliness, called the UCLA Loneliness Scale, in 1978 by Russell, et al. [14], and its revision in 1980 [15], along with other scales developed later [16,17], made it possible for further detailed studies to be conducted on loneliness, and for them to differentiate loneliness as an entity of its own, distinct from other conceptually related constructs [15,16,18,19]. In a study by Russell, et al. [15], the discriminant validity of the revised loneliness scale was tested against measures of psychosocial factors commonly associated with loneliness, namely negative affect (with depression and anxiety scales), low social risk taking (with measures of introversion/extroversion, assertiveness, sensitivity to rejection and self-esteem), lack of affiliative motivation (with measures of affiliative tendency and introversion/extroversion scales) and social desirability (with measures of social desirability and lying). The first three factors were found to be significant predictors for loneliness, but the fourth factor, social desirability, was not found a predictor of loneliness. However, taken together, these four factors could only predict less than half of the variance in loneliness scores. Furthermore, through a hierarchical regression analysis, the self-labeling loneliness index was still a significant stand-alone predictor of loneliness, after the variance attributed to the personality and mood factors was eliminated. The results of this study not only showcase clear evidence of the discriminant validity of the loneliness scale, but also introduce loneliness as a measurable distinct psychological experience, despite its correlation with other psychosocial factors [15].

It has long been suggested that loneliness has destructive consequences on health. Moreover, the various negative health outcomes associated with loneliness are not adequately predicted by the objective state of social isolation or a lack of social support, further distinguishing loneliness from constructs that are often mistakenly equated with it [20]. The deleterious effects of loneliness on the heart [21], leading to coronary heart disease and stroke [22], and on the brain, leading to dementia [23], and poor mental health [24], have been studied. Loneliness has also been related to metabolic syndrome [25], with depression as a correlating factor [26]. Furthermore, loneliness increases mortality of any cause [27], increasing the probability of death by 26% in reported loneliness [28]. This evidence strongly suggests that loneliness has a systemic effect on the body which eventually leads to its pathological consequences [29,30].

Three major pathological pathways have been suggested for the consequences of loneliness, namely poor health behaviors, heightened stress response and inadequate physiological repairing activity [31]. Through the first pathway, loneliness makes people more prone to behaviors such as smoking [21], less physical exercise [32] and even poorer sleep [33].

The latter two pathways, namely increased stress reactivity and disrupted physiological maintenance, have been subject to more specific inquiry. 

Stress and psychological stressors can be viewed from two perspectives; the exposure to them, and how they are perceived. Psychological stressors are physiologically perceived like threats, and can neurally activate the sympathetic nervous system (SNS) and the hypothalamic-pituitary-adrenocortical (HPA) axis. These two systems can then cause an inflammatory response [34]. Loneliness as a source of perceived stress, influences inflammatory processes through triggering a physiological stress cascade [35].

In an acute setting, SNS activation, at first, primes the immune system, as it mobilizes leukocytes into the blood. In addition, the stress-induced vagal withdrawal, lowers acetylcholine levels and allows for the inflammatory activity of tissue macrophages (developing an M2, rather than an M1, phenotype [36]) sitting in liver, spleen, heart, and the gastrointestinal tract, i.e., the vagally innervated reticulendothelial system [35]. Another effect of acute psychological stressors is the central release of interleukin-1 beta (IL-1β), directly activating the HPA axis, ultimately leading to the release of glucocorticoids, e.g., cortisol. In addition, brain IL-1β, also known as the leukocytic pyrogen [37], appears to be capable of causing manifestations of low-grade peripheral inflammation, e.g., elevated plasma cytokine levels and fever, in the case of severe stress [35]. To further explain the cases of severe or uncontrolled stress, as the adrenomedullary release of epinephrine follows, corticotropin-releasing hormone (CRH) not only activates the SNS, but also increases adrenocorticotropic hormone (ACTH) and adrenocortical steroids, manifesting into the consequences of autonomic stress responses. [38]

Glucocorticoids, on the other hand:

- Inhibit the release of brain IL-1β, containing and reducing inflammation
- Increase the production of IL-6, consequently inducing hepatic acute-phase reactants, e.g., C-reactive protein (CRP), minimizing cellular damage
- Increase the release of macrophage migration inhibitory factor (MIF) by macrophages, and consequently:
  - Reduce the sensitivity of immune cells to the anti-inflammatory effects of glucocorticoids,
  - Promote tumor necrosis factor-alpha (TNF-α) release, sustaining low-grade inflammation in the setting of chronic stress [35].

In the setting of chronic stress, e.g., loneliness, though there are higher levels of circulating glucocorticoids, many immune cells form an insensitivity towards them, which may be mediated by the production of MIF, ultimately leading to substantially lower anti-inflammatory effects of glucocorticoids and a greater susceptibility to inflammation and conditions correlated with it in the chronically stressed, e.g., lonely individuals [35].

Although the stress response is shared among humans, it varies in magnitude between individuals and thus people react differently to psychological stressors [30]. Lonely people are more prone to perceive regular events as stressful, in comparison with people who are not lonely [39]. This is evident in how differently social stressors can lead to higher levels of pro-inflammatory cytokines, in the blood and in the brain, relative to people’s personal and environmental circumstances [40]; such that lonely people show a higher increase, thus leading to a heightened inflammatory response [41]. This state of inflammation can be of survival advantage [42], as being isolated puts the individual in greater chance of getting attacked or wounded, with no protection from others, and the inflammation prepares the body to biologically endure any possible harm [43], which is favorable from an evolutionary standpoint [44]. In other words, inflammation, as present in loneliness and isolation, makes the individual more sensitive to negative experiences, seeing them as threats, and helps the individual potentially avoid them [44].

Reparative processes such as wound healing have been found faulty in the face of stress [45]. As touched upon earlier, sleeping, which is the most basic restorative behavior, is significantly affected in lonely people [33]; leading to a higher risk of cardiometabolic diseases [46].

With these three major pathways of pathogenesis, loneliness can do much harm; through metabolic processes mediated by immunologic factors leading to an inflammatory response. Functional activation patterns of genes have been studied as explanations to this observation.

Any social situation has different aspects, and that which is sensed (the physical aspect) and understood (the psychological aspect) in a social setting, leads to the activation of different gene modules, and thus results in the ultimate effect of an event on a person [47]. Different social settings precipitate distinctly appropriate activation of the human genome [48]. These different patterns of gene activation in different social settings were first observed in groups of leukocytes [49].

In the face of adverse life conditions, along with the appropriate genome activation that is specific to the encounter, a conserved general response is also activated [47]. This socially adjusted genome activation functions through the SNS (and β-adrenergic receptors), the HPA axis, and the glucocorticoid response, and is referred to as the conserved transcriptional response to adversity (CTRA) [50]. People with perceived loneliness, studied as an adverse life encounter, show the activation of CTRA, which causes an increased transcription of pro-inflammatory genes and a decreased expression of genes associated with antiviral responses, leaving the individual with inadequate defense against viruses [51]. Further solidifying the pattern of CTRA in loneliness, subjective isolation was found to be correlated with a lower expression of type I interferon (IFN) genes and certain transcripts in the synthesis of immunoglobulin G1 (IgG1), along with the increased expression of pro-inflammatory genes [34,47].

Taken together, we believe there are too many immunologic and metabolic manifestations associated with the construct of loneliness, and with the previous research showcasing loneliness as a distinct psychological experience [15,16,19], and a health determinant [21,52,53,54,55], we propose that loneliness, in and of itself, is not just a psychosocial phenomenon. It is also an all-encompassing complex of systemic alterations that occur with it, expanding it into a syndrome of events, concurrent with each other [56], and linked through a shared network of immunometabolic pathology. And although all of these changes may not happen at once in lonely individuals, the association between these factors and loneliness is individually significant, and as they share common pathways of pathology, we propose that their incidence and prevalence may covary over time, with the rise and fall of the feeling of loneliness.

This review aims to portray a detailed picture of loneliness as an immunometabolic syndrome, with its multifaceted pathology.

A categorized scheme of topics discussed in this review is presented in Table 1.

## 2. Immunologic Factors

To provide a framework for the changes in immunologic factors, we will delve into different categories of factors active in the immune system, including cytokines, immunoglobulins, measures of antiviral immunity, immune cell activity, and the genetic correlates of these factors.

As central components of the immune system, cytokines are soluble, low-molecular-weight proteins that immune or non-immune cells secrete, mediating and modulating the interactive functions and responses of the complex immune system. Cytokines are responsible for a wide array of functions, being an integral part of almost every biologic process [57,58]. These functions include regulation of host defense (non-specific response to infections, specific response to antigens, in addition to roles in vaccine efficacy and allograft rejection), cell-to-cell communication, tissue homeostasis, inflammatory reactions, embryonic development, and stem cell differentiation, pathogenesis of diseases, alterations in cognitive functions, and progression of the degenerative changes in aging [57,58].

With these many functions, cytokines encompass an assembly of biomolecules including inflammatory cytokines (interleukins (ILs), the family of tumor necrosis factors and adipokines), acute-phase reactants, chemokines, interferons, mesenchymal growth factors, colony-stimulating factors, and nerve growth factors [58,59,60].

### 2.1. Inflammatory Cytokines

Inflammation has been associated with loneliness in multiple studies [44,61,62], such that inflammatory responses to acutely stressful situations are heightened in loneliness [63]. This can be explained through the activation of CTRA, as previously mentioned [51]. CTRA can also cause a perception of loneliness, therefore the connection between inflammation and perceived social stressors is reciprocal [64]. It was suggested that stress, along with troubled interpersonal relations, could synergistically cause increased inflammatory responses [65]. An example of this is present in the face of biologically challenging stressors, like endotoxin [65].

#### 2.1.1. Endotoxin Trials

In a trial of endotoxin on healthy participants, the association between sensitivity to social disconnection and pro-inflammatory reactions was studied. This sensitivity to social disconnection was portrayed as a composite model with loneliness, sensitivity to rejection, fear of negative judgment, and anxious attachment scores included. In response to endotoxin, those who were more socially sensitive to disconnection according to the composite model had heightened pro-inflammatory profiles with a CTRA prominence, up-regulated inflammatory gene expression, and higher levels of TNF-α and IL-6 [43].

In another study on this topic, breast cancer survivors would go through a social stress test and be tested for loneliness, while lipopolysaccharide (LPS, a bacterial endotoxin) was introduced to cultures of their peripheral blood mononuclear cells (PBMCs), collected before and after the acute stress, and cytokine production was measured. In lonelier participants, a significant elevation in IL-6 and interleukin-1 beta (IL-1β), along with a non-significant elevation in TNF-α was observed, confirming the pro-inflammatory phenotype of loneliness [66].

#### 2.1.2. IL-6

IL-6 levels have been found positively associated with loneliness in multiple studies [43,66,67,68,69,70,71,72,73]. The cytokine has been described to have pleiotropic activity, as it promotes the production of acute-phase reactants such as CRP, fibrinogen, serum amyloid A and hepcidin in hepatocytes, and inhibits albumin synthesis [74]. IL-6 is also active as an antibody production stimulant and effector T-cell development regulator and is therefore essential in the functionality of acquired immunity. Another area of action for IL-6 is in the multiplication and differentiation of different non-immune cells in the body. With Its many functions, an imbalance of IL-6 production can be detrimental to health [74]. Moreover, elevated IL-6 levels have been associated with many age-related diseases [75].

#### 2.1.3. TNF-α

TNF-α is a pro-inflammatory cytokine that has different roles in lymphoid activation and humoral immunity against infectious agents [76], as well as regulating the adhesion of leukocytes to epithelium through the synthesis of adhesion molecules [77]. TNF-α also participates in processes of vasodilatation and edema formation, blood coagulation, oxidative stress in the presence of inflammation, and the induction of fever [77]. Loneliness and TNF-α levels have been found positively correlated [43,66].

#### 2.1.4. IL-2 and IL-10

IL-2 [78] and IL-10 [79] levels are also affected by loneliness. In a study on the correlation between the cytokine profile of breast cancer survivors and their psychosomatic composite model, which was composed of test scores regarding anxiety, perceived stress, loneliness, depression, sleep quality, daytime sleepiness, and fatigue, IL-2 and IL-10 were found to be the most important features in the model and therefore associated with loneliness by proxy [80].

### 2.2. Growth Factors

#### 2.2.1. VEGF

Inadequate social support and worse cancer outcomes have been correlated. In a study aiming to examine proangiogenic cytokines with regard to social support in individuals with colon and rectum tumors, implicit loneliness was associated with increased levels of vascular endothelial growth factor (VEGF) in the tumors; this suggests that VEGF could be the mediator between loneliness and a poorer prognosis in cancer, by promoting angiogenesis [81]. Further studies directly examining the effects of loneliness are suggested.

#### 2.2.2. IGF-1

Insulin-like growth factor 1 (IGF-1) regulates the progression of the cell cycle, cell death, and protein translation. It has also been found associated with immune coordination through influencing certain aspects of inflammation, thymus development, and the differentiation of immune cells, namely T and B lymphocytes, monocyte-macrophages, and other immune cells [82]. Loneliness was inversely associated with serum levels of IGF-1 [83]. Further studies to reinforce the results of the cited study are suggested.

#### 2.2.3. GM-CSF

GM-CSF levels have been found associated with loneliness [80], and this association can be explained by the distinct way that CTRA acts in the face of adversity, which is manifested through the SNS. The SNS can regulate hematopoietic mechanisms using β-adrenergic signals. These signals increase the transcription of granulocyte-macrophage colony-stimulating factor (GM-CSF), which is a myelopoietic growth factor, and thereby boost the differentiation of monocytes. This results in an increased number of immature pro-inflammatory monocytes which are then responsible for the observed transcriptional response to adversity [84].

### 2.3. Acute-Phase Reactants

#### 2.3.1. CRP

CRP, which is considered the prototypic acute-phase reactant, is a marker of inflammation and infection. It activates the complement system, recognizes pathogens, and interacts with F_cγ_ receptors, and is therefore active in both innate and adaptive immunity. CRP has been associated with the induction of atherosclerosis, as well as having an anti-inflammatory function in autoimmunity [85]. CRP levels were found positively associated with loneliness [42,70,72,86].

#### 2.3.2. Fibrinogen

Fibrinogen is an essential factor in blood coagulation. It was found to increase autoimmune reactions, through the presentation of antigens and the release of chemokines [87]. Fibrinogen was found both positively [70,88], and negatively [89], associated with loneliness. In other studies, no association was found with loneliness and fibrinogen [67,90]. As one of the studies denoting no association between loneliness and fibrinogen was a systematic review [67], their results could offer a more comprehensive view. We believe the observed discrepancies could be due to inadequate sample sizes, along with greater individual differences in loneliness than previously thought.

#### 2.3.3. Ferritin

Ferritin is an iron storage protein and an inflammatory marker, correlated with cell damage, oxidative stress, and the incidence or severity of several diseases [91]. Ferritin levels have been found to increase at the onset of loneliness in men, and to decrease with long-term loneliness in older women [86]; however, as we didn’t find any further evidence on this topic, future studies on the effect of loneliness on ferritin levels are suggested.

### 2.4. Chemokines

#### 2.4.1. MCP-1

Monocyte chemotactic/chemo-attractant protein-1 (also known as C–C motif chemokine ligand 2, MCP-1/CCL2) is a key chemokine in the regulation of monocyte and macrophage migration and infiltration and has been associated with inflammatory diseases like atherosclerosis and rheumatoid arthritis [92]. In studies on the immune response to standardized mental stress, lonelier women [68], along with lonelier people with type 2 diabetes [93], showed higher levels of MCP-1, which emphasizes the role of inflammation in the manifestation of the consequences of loneliness [68].

#### 2.4.2. MIF

Macrophage Migration Inhibitory Factor (MIF) has also been shown to act as a key regulator of inflammatory cell recruitment and atherogenesis by displaying chemokine-like activity [94]. In a study on university undergraduates who scored high on loneliness and depression tests, those with higher scores had 40% higher plasma MIF levels in the face of a public speaking test, in comparison with the ones with lower scores. Therefore, MIF can be considered a neuroimmune factor, mediating the association between depressive symptoms and inflammation [95], which should be further studied in this regard.

### 2.5. Immunoglobulins

Loneliness can negatively affect the proper activity of humoral immunity. This has been observed in immunoglobulin studies. Lonelier elders had much lower levels of IgG, IgA, and IgM, along with a higher mortality rate over a five-year period [96]. In first-year medical students, a significant increase in total plasma IgA was observed on the first day of final exams, compared to a month prior [97].

Mucosal immunity is another arena for the immune functions of immunoglobulins, which if impaired due to stress, could leave the individual susceptible to infection [98]. Experiencing stress, either subjective or objective, was found associated with decreased salivary IgA levels. This negative association may be mediated through specific personality traits like loneliness [99]. To further study the effect of loneliness and perceived stress on secretory immunoglobulin A (S-IgA), its subclasses (S-IgA1 and S-IgA2), and the associated transporter molecule, secretory component (SC), salivary levels of all these molecules were measured in university students. IgA-1 levels were reduced in heightened perceived stress. On the other hand, SC levels were increased in loneliness; meaning psychological stress negatively affected mucosal immunity by relative depletion of immunoglobulins, leaving the enhanced immunoglobulin transport capability disproportionate to its demand [98]. The S-IgA to albumin (Alb) ratio was found to be decreased in the presence of negative emotional states and difficult physical straining states such as restrictions in diet, loss of body mass, and in the case of upper respiratory tract infections, proving to be a useful indicator of training severity [100].

### 2.6. Antiviral Immunity

It is much less likely to be exposed to a socially borne pathogen if an individual is isolated. In addition, given the fact that they will not have protective company around, it is more likely for a lonely person to be physically hurt or wounded, which can be a bigger threat to immediate survival in comparison to a virus they would hardly encounter. Therefore, needing an inflammatory response in order to heal or even to live would be considered more vital than the need to fight a virus they have low chances of encountering, making inflammation a priority over antiviral response in the context of reduced affiliative behavior [44]. Congruent with this perspective from evolution, loneliness can also lead to less robust antiviral immunity [44], such that as mentioned earlier, subjective isolation is correlated with a lower expression of type I interferon (IFN) genes and certain transcripts in the synthesis of immunoglobulin G1 (IgG1) [34,47].

#### 2.6.1. Interferons

Studies have shown lower levels of IFNs in lonely people [64,79]. In a transcriptome study aiming to better understand the immune effects of CTRA in loneliness, both type I and type II IFNs were down-regulated in perceived social isolation, expressed in a rhesus macaque model [64]. The interferon response against viruses was also found impaired, for example, against the simian immunodeficiency virus (SIV) [64].

In addition to suppression of immunity, loneliness can also cause a dysregulation in immune responses. To further explain this matter, lonely people have a harder time subjectively adjusting to a stressful situation, thus challenging settings could affect the lonelier individual more severely. It was observed that IFN-γ, which is a type 1 cytokine, was decreased and IL-10, which is a type 2 cytokine, was increased in lonelier individuals in stressful circumstances. Therefore, the type 1 and type 2 cytokine dynamic in lonely people shifts toward type 2 in times of stress, which can potentially lead to the emergence of more type 2 mediated immune presentations, such as higher rates of viral infection, autoimmunity, asthmatic or allergic reactions and reactivation of latent viruses in periods when the individual is exposed to high-stress environments [79].

#### 2.6.2. Antibody Response against Viral Infection

The compromising consequences of loneliness on antiviral immunity extend to the antibody response to viruses. Antibody titers against certain viruses [99], namely Cytomegalovirus (CMV) [101], Epstein-Barr virus (EBV) [101], Herpes simplex virus 1 (HSV-1) [101,102] have been found elevated in loneliness.

Latent virus reactivation can provide an important insight into the dysregulation of the immune system. Antibody titers against CMV, which is a herpesvirus, were higher among lonelier breast cancer survivors. The higher antibody level in lonely individuals was also associated with a heightened “pain, depression and fatigue” symptom cluster, which can be a source of distress in cancer survivors [103].

The mediating role of social integration on the effect of a severe environmental source of stress, on the feeling of loneliness and immune fitness was investigated in those with a human immunodeficiency virus (HIV) infection. Antibody titers against human herpesvirus 6 (HHV-6) in this population were higher among those who were lonelier and had less social support, which indicates the compromised fitness of cellular immunity in controlling the viral infection [104].

#### 2.6.3. Antibody Response against Vaccines

In a study measuring antibody response to influenza immunization with regard to social integration, having a small social network, and higher levels of loneliness caused a poorer reaction to immunization. Moreover, the weakest antibody response to the vaccine was observed in those who were lonelier and had smaller social networks. This loneliness-antibody response axis was found to be mediated by stress levels [105].

#### 2.6.4. Behavioral Immunity against Viruses of Social Stigma

The stigma around infection with viruses like HIV, hepatitis C virus (HCV), and the severe acute respiratory syndrome coronavirus 2 (SARS-CoV-2), can escalate the isolation and loneliness that already comes with the concept of illness. To emphasize this matter, it should be pointed out that the mere diagnosis of HCV can lead to feelings of social isolation [106]. Aging with HIV has also been associated with feelings of loneliness [107]. These feelings of isolation can affect the individual’s attitude toward seeking and committing to treatment. For example, commitment to antiretroviral therapy for an HIV infection has been found to be negatively affected by the internalized stigma of the individual toward being infected and has therefore led to suboptimal adherence to treatment [108,109,110]. Even before being infected with the virus, in the case of SARS-CoV-2, lonelier people were less committed to taking the necessary preventative measures against infection [111]; therefore immune behaviors against the contraction of viruses can be negatively affected by loneliness, putting the lonely individual at higher risk of infection [111], which will then come with social stigma [112], feeding into the vicious cycle of loneliness and infection with stigmatized viruses. 

### 2.7. Immune Cells’ Number and Activity

Loneliness can affect the proper activity of cellular immunity and natural killer (NK) cells [113]. In a study on middle-aged adults, lonelier participants exhibited smaller increases in NK cell numbers in response to mental stress tests [88]. Furthermore, those who are lonelier exhibit less NK cell activity, than those who are more connected socially; thus leading to a suboptimal antiviral and antitumor defense in isolated individuals [97,114]. Mitogen stimulation by lymphocytes is also reduced in loneliness [99]. 

Social integration can also affect immune cell numbers. In a study on adults aged 50 or older, more social engagement and not living alone were associated with a lower white blood cell (WBC) count [83]. Perceived social isolation, whether chronic or variable at different times, has been found associated with higher percentages of circulating monocytes [84,115], which can be explained through CTRA. As mentioned before, CTRA causes a shift in patterns of gene expression [51], which was found to stem from myeloid lineage immune cells, namely monocytes and dendritic cells [116]. Studies have confirmed the mediating role of monocytes in the transcriptional changes of CTRA [117,118]. Furthermore, within the increased percentage of monocytes, the immature, pro-inflammatory CD14^++^/CD16^−^ classical monocyte transcriptome is selectively up-regulated in the context of loneliness and thus this subpopulation of monocytes is probably responsible for originating the CTRA pattern of transcriptome shift [64].

Other factors involved in immune functions can be affected by social settings as well. For example, loneliness was negatively associated with soluble intercellular adhesion molecule-1 (sICAM-1) [72], which is also a counter-receptor for the lymphocyte function-associated antigen (LFA-1) [119].

### 2.8. Genetic Correlates of Immune Regulation

With regard to the aforementioned social regulation of gene expression, social integration differs from its lack in the expression levels of 209 transcripts in PBMCs, with a net reduction of gene expression in lonely people [116]. The up-regulation of genes responsible for immune activation (e.g., molecules promoting the progression of cell cycle and pro-inflammatory cytokines), transcription control, and cell growth/differentiation (e.g., chromatin structure regulators, enzymes in the biosynthesis of nucleotide and protein, factors in cytoskeletal remodeling, RNA processing, and nuclear export factors), along with the complementary down-regulation of genes involved in cell cycle inhibition and apoptosis, and the anti-proliferative transforming growth factor beta (TGF-β), together, lead to an overly proliferative immune phenotype in lonely individuals [42].

In contrast to this activated face of immunity, certain aspects of the immune response are down-regulated in loneliness, such as genes in type I interferon response, genes involved in immunoglobulin light, joining (J), and heavy region chains, markers of B lymphocyte function and transcription factors responsible for B cell maturation [42].

Further differentiating the effects of social isolation and loneliness, physical (objective) isolation has been found associated with reduced levels of antibody synthesis gene expression, which can be explained by the reduced exposure to microbes that are socially transmitted, and perceived (subjective) isolation has been found associated with an elevation in pro-inflammatory gene expression and a reduction in genes related to IgG1 and type I IFN synthesis [34,47].

An overview of the immunologic factors is presented in Table 2.

## 3. Metabolic Factors

### 3.1. Markers of HPA Function

#### 3.1.1. Regulation of Glucocorticoids in Loneliness

Stress is a significant influencing factor in human life, affecting the individual through immune, endocrine, and cardiovascular systems as the body tries to rise up to challenges and successfully endure them, whether the challenges are psychological or environmental. This stress response, through the SNS and HPA axis, causes the release of catecholamines and glucocorticoids, namely epinephrine and cortisol, respectively. The increase in these hormones can help the individual adapt to acutely demanding circumstances, but if persisting for longer periods, they can be detrimental to health [123]. In a study on young adults, three different durational correlates of loneliness and their effects on the HPA axis were examined, namely trait, momentary and daily correlates [124], highlighting the fact that loneliness can be examined as both an acute or chronic phenomenon. Prolonged or fluctuating exposure to stressful stimuli causes chronic or periodic changes in neural and hormonal responses, respectively; which result in a “biological cost” for the body with the resultant wear and tear, referred to as the allostatic load [123,125].

Social adversity, e.g., loneliness, which can be a source of prolonged stress, activates the HPA axis, causing an elaboration of cortisol which reduces the allostatic negative feedback, causing an allostatic overload. Consequently, the glucocorticoid receptor gene (Nuclear Receptor Subfamily 3 Group C Member 1, NR3C1) is down-regulated [126], leading to functional glucocorticoid desensitization [64], which is also correlated with the CTRA pattern of gene expression [50], providing an additional transcriptional etiology for this phenomenon in the context of loneliness.

To further describe the regulation of glucocorticoids in loneliness, the association between glucocorticoids and MIF should be addressed. MIF, which was discussed earlier, is released when physiological glucocorticoid levels are low. However, MIF subsequently reduces immune cell sensitivity to anti-inflammatory effects of glucocorticoids. This antagonistic interdependent behavior between MIF and glucocorticoids can function as a balancing factor for the effects of glucocorticoids on immune reactions [127,128]. Moreover, MIF and ACTH are secreted by the same group of anterior pituitary cells, which can be considered an anatomical reflection of their close functional association [129]. As MIF levels were higher in lonely people in the face of stress, suppression of the glucocorticoid response is expected in loneliness [95], which is congruent with the observed glucocorticoid desensitization [64]. Therefore, MIF can be considered a communicating factor between the immune and metabolic aspects of loneliness.

#### 3.1.2. Counter-Regulation of the Stress Circuitry in Loneliness

With our earlier mentions of the HPA axis and its pro-stress hormones such as glucocorticoids and ACTH, the picture of the HPA axis in loneliness will not be complete without discussing its associated neuromodulators. As mentioned before, chronic loneliness causes an elevation of mean salivary cortisol levels, suggesting both an increased activation of the HPA axis and a rise in the production of CRH [130], but these elevations and imbalances in circulating stress hormones [131] should be buffered. Keeping the complex stress circuitry in balance requires specific regulatory pathways and it is important to mention the different mechanisms the stress circuitry has to counter-regulate. The significance of this matter can be highlighted in two ways, as these stress counteracting mechanisms can: one, lead the way to the treatment or prevention of stress-related neuropsychiatric disorders, and two, showcase the expression of individual differences in resilience or vulnerability to stress [132].

To further explain one of these counter-regulatory mechanisms, in the pro-stress vs. anti-stress mechanism, the pro-stress neuromediators, e.g., CRH, are counteracted by the stress-elicited activation of anti-stress neuromodulators, e.g., neuropeptide Y, endocannabinoids, urocortins, and endogenous opioids, e.g., enkephalin [133,134,135,136,137,138,139]. The pro- and anti-stress mechanism, or the stress-buffering hypothesis, states that loneliness upregulates stress responses, heightening neuroendocrine and sympathetic activity [140]. Changes in the levels of endogenous opioids and oxytocin (as counteracting mediators) have been suggested as underlying factors for some of these observed consequences, in arising animal and human studies [140]. However, in the articles we found on this topic, the animal studies mostly observed the effects of social isolation [141,142,143,144], or loss of a bonded partner [145] on the CRH system, endogenous opioids or oxytocin, and not the effect of the perceived entity of “loneliness” [146], and the human studies were mostly on the effect of depression on endogenous opioids (although including “alone or lonely subscales” and reporting a lower µ-opioid receptor binding potential in major depressive disorder [147]), and thus further human studies specifically aimed at loneliness are suggested in this area. 

#### 3.1.3. Measures of Cortisol in Loneliness

Cortisol levels rise and fall steadily in a diurnal pattern, with the highest levels about 30 min after waking, and the lowest at midnight [148]. Specific measures of cortisol can capture the diurnal pattern and changes in cortisol. Waking cortisol is usually expressed in its salivatory level. Cortisol awakening response (CAR) captures the elevation in cortisol output, from immediately after waking to 30 to 45 min after waking. The diurnal slope (DS), captures the “slope” of changes in cortisol from its peak (at wake) to a level representing its nadir [149].

Loneliness was associated with an attenuated CAR [124,149]. Regarding the DS, it was found to be either flatter [124], or steeper [149,150] in loneliness. To explain this discrepancy, Lai, et al. stated they found the steeper DS was primarily a result of higher waking cortisol levels [149], as opposed to the other studies primarily basing their results on nadir levels; and the other studies used different measures of loneliness, which could also be the cause of this discrepancy [149]. The overall secretion of cortisol (reflected in the area under the curve with respect to ground, AUC_G_) was increased in loneliness [149]. As elevated diurnal cortisol levels and a blunted CAR have been correlated with poorer health outcomes in previous literature, further research on the pathways connecting loneliness to pathological endpoints through the effects of elevated cortisol is warranted [149].

Cortisol output post-stress was decreased in loneliness, reflecting the disturbances in stress reactivity in loneliness [93].

Salivary waking cortisol levels were found to be either increased [88,124,149] or decreased [68,69,95] in loneliness, and some studies didn’t find a correlation between the two [21,79]. We believe these differences could be due to insufficient sample sizes and/or different controlling for covariates. To further explain these discrepant results, Lai, et al. suggest that the use of different measures for loneliness in the studies, together with the findings of a genome-wide study [151] denoting loneliness to be a trait with polygenic architecture and only modestly heritable, taken together, imply that trait loneliness has a more heterogeneous phenotype than previously thought of [149].

As exposure to stress causes a release of cortisol, it can be considered a marker of psychological stress [148], e.g., loneliness, and the observed associations between loneliness and measures of cortisol is evidence of that. Salivary cortisol levels have been commonly used in this regard, although it should be noted that these levels only portray an indirect measure of the involvement of psychological triggers to the HPA axis and do not reflect ACTH levels directly, as different factors in the HPA axis can affect the production of cortisol [148]. In a study on overall and momentary solitude, HPA function and affect in different age groups, salivary cortisol levels, and dehydroepiandrosterone sulfate (DHEAs) as markers of the HPA axis were measured. Both momentary and overall solitude lead to increased average salivary cortisol and DHEAs levels. Overall solitude led to negative affective and systemic repercussions, which could be detrimental to health in the elderly. However, momentary solitude was found associated with some positive affective influences, and was less negatively impactful on older people. It appears that aging can soften the effects of momentary solitude on affect, though it comes with a systemic cost [152].

### 3.2. Markers of Glycemic Control

Loneliness has been associated with type 2 diabetes mellitus (DM) [153,154], hyperglycemia [26], and higher hemoglobin A1c (HbA1c) [155,156] and fasting blood sugar (FBS) [25] levels. Although some studies have found no significant relation between HbA1c and loneliness [157,158,159]. Social under-stimulation was associated with higher HbA1c levels [160]. Loneliness was also found associated with poorer glycemic control in Type 2 DM [161].

### 3.3. Markers of Lipid Metabolism

Higher triglyceride [26,162,163] and lower high-density lipoprotein (HDL) cholesterol (Chol) levels [26,162] were found in lonely people. However, total Chol, low-density lipoprotein (LDL) Chol, and non-HDL Chol levels were not correlated with loneliness [157]. In addition, lonely people had lower erythrocyte membrane proportions of Eicosapentaenoic Acid (EPA) and Docosahexaenoic Acid (DHA), which are omega-3 fatty acids [164].

### 3.4. Indices of Weight Control and Body Composition

Body mass index (BMI), as a widely used index of weight control and body composition, was increased in loneliness [78,156,163,165]. Obesity as expressed in central obesity, higher waist circumference, non-abdominal obesity or higher body fat, was increased in loneliness [25,162,163,166,167,168,169]. Eating alone was associated with central obesity [170]. Moreover, lonely people were more prone to increased food consumption [102] and higher intake of sugary beverages [171], which reflects the disturbed glycemic control that was mentioned earlier. Appetite-related hormones such as ghrelin and leptin were also found dysregulated in loneliness [102], which could potentially explain the observed dietary behaviors.

With the social and physiological changes that come with aging, a contrasting picture of weight control was observed in loneliness, as it was associated with anorexia of aging [172] and insufficient dietary intake and trace metals in the elderly [173,174,175,176].

### 3.5. Meeting the Criteria for Metabolic Syndrome

With the aforementioned indices of body composition, glycemic control and lipid profile, a cumulative metabolic burden [156,166] leading to a diagnosis of metabolic syndrome seems inevitable and has been observed in loneliness [25,26,162]. The overall prevalence of metabolic disorders has also been increased with loneliness [163].

### 3.6. Elements of Cardiovascular Function

Loneliness is associated with hypertension [26,155], with both increased systolic [21,69,78,157,177] and diastolic [21,88] blood pressures. Other elements of cardiovascular (CV) function such as heart rate, cardiac output, and cardiac contractility were found to be reduced, while total peripheral resistance was increased in loneliness [21]. Autonomic cardiac control was decreased as well [178].

As a cumulative result of these manifestations, rates of CV diseases were increased in loneliness [153,163,179]. Rates of coronary heart disease (CHD) and stroke [22], along with CV mortality were also increased in loneliness [180], even in those with stable CHD who were receiving medicine as secondary prevention [181]. New York Heart Association (NYHA) class was also found to be raised in lonelier heart failure patients [182].

### 3.7. Markers of Cognitive Function

Loneliness is associated with an increased risk for the onset of dementia [23,183]. Cortical amyloid burden, which was studied as a biomarker of Alzheimer’s disease, was elevated in lonelier cognitively normal elders, such that this elevation was 7.5 times more likely to be observed in those who were lonely [183]. Amyloid-beta (Aβ) levels, specifically Aβ-40 and Aβ-42 levels, and Tau protein levels were found to be associated with loneliness [80].

Perceived social isolation can be behaviorally defined in both humans and animals, and although certain aspects of this perception may be unique to humans, it is nevertheless shared across different species and has been associated with lower brain-derived neurotrophic factor (BDNF) expression [184].

Regarding structural brain correlates of loneliness in the elderly, smaller volumes of gray matter were found in three clusters of brain structures that have been associated with processing emotions, namely in the left cerebellum, left amygdala-anterior hippocampus and the left posterior parahippocampus. Loneliness was also associated with latent factors of the hippocampus and amygdala [185]; however, as the nature of these associations between brain structure and loneliness is cross-sectional, no conclusions on the temporal direction of these changes, let alone causality between the two, can be made [185].

### 3.8. Elements of Mental Health

Mental health outcomes are negatively affected by loneliness [24,105,163]. A higher number of hassles, along with trouble adjusting to them, are positively correlated with loneliness [79]. 

The most commonly observed mental health outcome of loneliness is depression [26,88,182,186]. Loneliness can also predict depressive symptoms [20,187]; but it is important to mention that depressive symptomatology was found not to be a predictor of loneliness, denoting a one-way relationship between the two, highlighting the temporal association between these phenomena [20]. Furthermore, through constructing cross-lag models, it was determined that the association between loneliness and increases in depressive symptoms is not merely a reflection of, and cannot be reduced to, the effect of covariates such as demographic factors, objective social isolation, perceived stress, exposure to life stressors, negative affectivity or social support [20].

As to why some prior studies found a reciprocal relationship between depressive symptomatology and loneliness, evidence suggests that this can be explained by the masking quality of the objective exposure to life stressors, on the predictive value of depressive symptoms on changes in loneliness; and although this masking quality is small and warrants replication, it still provides some perspective [20]. It is important to mention that this masking effect was not reciprocal, meaning exposure to life stressors did not mask the effect of loneliness on depressive symptoms. The source of this masking effect was identified to be the presence of a psychiatric diagnosis when life events were held constant, and therefore in those without a psychiatric diagnosis, depressive symptomatology could no longer predict changes in loneliness while holding life events constant [20]. Although those with a psychiatric diagnosis in the sample were too few for a reliable estimate, it was still inferable that depressive symptoms can more strongly affect changes in loneliness in those with a psychiatric diagnosis, as these people also reported more stressful life events, possibly explaining the observed differences. Additional research is required to solidify this hypothesis [20].

In a study on loneliness in schizophrenia, it was more common for people with schizophrenia to feel lonely than it was for healthy controls, whether or not they were objectively isolated. The lonelier they were, the more likely it was for them to be diagnosed with drug abuse or drug dependence, have a higher number of used drugs, and have hypertension or HbA1c dysregulations. Loneliness was also associated with metabolic syndrome in this population [155].

Sleep quality was negatively impacted by loneliness [21,46,88,105]. Health behaviors were also negatively affected in loneliness [166,177,188], with increased use of recreational drugs [21], and lower physical activity [189], which could all negatively affect mental health.

### 3.9. Genetic Correlates of Metabolic Regulation

As explained earlier, loneliness as a state of perceived social isolation can activate the HPA axis. The resultant elaboration of cortisol reduces the allostatic negative feedback, causing an allostatic overload. As a result, glucocorticoid receptor gene (NR3C1) expression is reduced [126], leading to functional glucocorticoid desensitization [64], which can also be explained by the CTRA pattern of gene expression [50]. Moreover, a reduction in the expression of genes involving anti-inflammatory glucocorticoid response elements (GREs) [42,190], and an increase in the expression of genes involving response elements of pro-inflammatory NF-κB/Rel (Nuclear Factor Kappa-light-chain-enhancer of Activated B Cells/Rel homology domain) transcription factors have been observed in lonely people, which both ultimately support the inflammatory profile of gene expression in loneliness. However, this reciprocal alteration in the dynamic between pro- and anti-inflammatory gene regulation was not correlated to plasma cortisol levels [42], which reflects the fact that multiple pathways influence this phenomenon.

Markers of oxidative stress are also up-regulated in loneliness [190]. In an animal model of social isolation an up-regulation of genes associated with DNA methylation and a broad spectrum of global epigenetic changes was observed, which can influence patterns of gene expression in the isolated animals and thus affect their phenotype [191]. Further human studies with a focus on loneliness are suggested in this area.

An overview of metabolic factors is presented in Table 3.

## 4. Conclusions

With the many immunologic and metabolic consequences of loneliness, it clearly asserts itself as more than just a fleeting feeling or a negative thought. Loneliness not only affects our psychological state, but also takes hold of almost all systemic regulatory pathways in our body and poses serious threats to our physical and mental well-being. Therefore, it seems reasonable to introduce loneliness as an “immunometabolic syndrome”, as the term reflects the multifaceted pathological manifestations of this perceived state of social isolation.

## Figures and Tables

**Table 1:** Topics discussed in this review.

Headings		Subheadings	Examples
Immunologic factors	*1*	*Inflammatory cytokines*	IL-6, TNF-α, IL-2, IL-10
	*2*	*Growth factors*	VEGF, IGF-1, GM-CSF
	*3*	*Acute-phase reactants*	CRP, Fibrinogen, Ferritin
	*4*	*Chemokines*	MCP-1, MIF
	*5*	*Immunoglobulins*	IgA, IgG, IgM
	*6*	*Antiviral immunity*	
		6.1. Interferons	Type 1 and 2
		6.2. Antibody response against viral infection	CMV, EBV, HSV-1, HHV-6
		6.3. Antibody response against vaccines	Influenza vaccination
		6.4. Behavioral immunity against viruses of social stigma	HIV, HCV, SARS-CoV-2
	*7*	*Immune cell number and activity*	WBC count, NK cell activity
	*8*	*Genetic correlates of immune regulation*	
Metabolic factors	*1*	*Markers of HPA function*	
		1.1. Regulation of glucocorticoids in loneliness	
		1.2. Counter-regulation of the stress circuitry in loneliness	
		1.3. Measures of cortisol in loneliness	
	*2*	*Markers of glycemic control*	HbA1c, FBS, T2DM
	*3*	*Markers of lipid metabolism*	Tg, Chol, HDL:Chol
	*4*	*Indices of weight control and body composition*	BMI, Obesity, Central adiposity, Anorexia
	*5*	*Meeting the criteria for metabolic syndrome*	
	*6*	*Elements of cardiovascular function*	BP, HR, CO, TPR
	*7*	*Markers of cognitive function*	BDNF, Amyloid beta
	*8*	*Elements of mental health*	Depression, Schizophrenia, Sleep quality
	*9*	*Genetic correlates of metabolic regulation*	NR3C1, NF-κB/Rel

Abbreviations. IL: Interleukin, TNF-α: Tumor Necrosis Factor-alpha, VEGF: Vascular Endothelial Growth Factor, IGF-1: Insulin-Like Growth Factor 1, GM-CSF: Granulocyte-Macrophage Colony-Stimulating Factor, CRP: C-Reactive Protein, MCP-1: Monocyte Chemotactic/Chemo-Attractant Protein-1, MIF: Macrophage Migration Inhibitory Factor, Ig: Immunoglobulin, CMV: Cytomegalovirus, EBV: Epstein-Barr Virus, HSV-1: Herpes Simplex Virus 1, HHV-6: Human Herpesvirus 6, HIV: Human Immunodeficiency Virus, HCV: Hepatitis C Virus, SARS-CoV-2: Severe Acute Respiratory Syndrome Coronavirus 2, NK Cell: Natural Killer Cell, HPA: Hypothalamic-Pituitary-Adrenocortical, T2DM: Type 2 Diabetes Mellitus, HbA1c: Hemoglobin A1C, FBS: Fasting Blood Sugar, Tg: Triglycerides, Chol: Cholesterol, HDL: High-Density Lipoprotein, BMI: Body mass index, BP: Blood Pressure, HR: Heart Rate, CO: Cardiac Output, TPR: Total Peripheral Resistance, BDNF: Brain-Derived Neurotrophic Factor, NR3C1: Nuclear Receptor Subfamily 3 Group C Member 1, NF-κB/Rel: Nuclear Factor Kappa-light-chain-enhancer of Activated B Cells/Rel Homology Domain.

**Table 2:** An overview of the immunologic factors investigated in relation to loneliness.

Immunologic Factors		Relation to Loneliness		
		+	−	No/NS
Inflammatory cytokines	IL-6	[43,66,67,68,69,70,71,72,73]		[78,81,93,120]
	TNF-α	[43,66]		[66]
	IFNs		[64,79]	
	IL-1α			[121]
	IL-1β	[66]		
	IL-10	[79]		
	IL-1RA	[68]		[93]
	IL-2			[78] (NS)
Growth factors	VEGF	[81]		
	IGF-1		[83]	
	GM-CSF			[80] (NS)
Adhesion molecules	sICAM-1	[72]		
Acute-phase reactants	CRP	[42,70,72,86]		[67,89,97]
	Fibrinogen	[70,88]	[89]	[67,90]
	Ferritin		[86]	
Chemokines	MCP-1	[68,93]		
	MIF	[95]		
Immunoglobulins	Plasma IgA	[97]	[96]	
	Salivary IgA	[99]		[97]
	Plasma IgG		[96]	[97]
	Plasma IgM		[96]	[97]
Antibody against viruses	CMV	[101,103]		
	EBV	[101]		[103]
	HSV-1	[101,102]		
	SIV		[64]	
Antibody response against vaccines	Influenza immunization		[105]	
	Low dose rDNA hepatitis B vaccine			[122]
Immune cell activity	NK cell activity	[88]	[97,99]	
	Lymphocyte functions		[99]	
Genetic correlates of immune regulation	Genes supporting mature B lymphocyte function		[42]	
	Genes in type I interferon response		[42]	
	Genes involved in immune activation, transcription control, and cell proliferation	[42]		

Abbreviations. +: positive association, −: negative association, No: No relation, NS: non-significant relation, IL: Interleukin, TNF-α: Tumor Necrosis Factor-alpha, IL-1RA: Interleukin-1 Receptor Antagonist, VEGF: Vascular Endothelial Growth Factor, IGF-1: Insulin-Like Growth Factor 1, GM-CSF: Granulocyte-Macrophage Colony-Stimulating Factor, sICAM-1: Soluble Intercellular Adhesion Molecule-1, CRP: C-Reactive Protein, MCP-1: Monocyte Chemotactic/Chemo-Attractant Protein-1, MIF: Macrophage Migration Inhibitory Factor, Ig: Immunoglobulin, CMV: Cytomegalovirus, EBV: Epstein-Barr Virus, HSV-1: Herpes Simplex Virus 1, SIV: Simian Immunodeficiency Virus, rDNA: Recombinant DNA, NK Cell: Natural Killer Cell.

**Table 3:** An overview of metabolic factors investigated in relation to loneliness.

Metabolic Factors		Relation to Loneliness		
		+	−	No/NS
Markers of HPA function	Cortisol output post-stress		[93]	[95]
	Salivary waking cortisol levels	[88,124,149]	[68,69,95]	[21,79]
	Plasma cortisol	[105,124,192]		
	Cortisol awakening response (CAR)	[124,149]		
	Cortisol diurnal slope (DS)	[149,150]	[124]	
	Cortisol AUC_G_	[149]		
	DHEAs			[78]
Markers of glycemic control	T2DM	[153,154]		
	Hyperglycemia	[26]		
	HbA1c	[155,156]		[157,158,159]
	FBS			[25](*p* = 0.07)
	AKT activity	[193]		
	“Glycemic success” in T2DM		[161]	
	fear of hypoglycaemia in adults with T1DM			[194]
Markers of lipid metabolism	Tg	[26,162,163]		[25,157]
	Total Chol			[157]
	HDL Chol		[26,162]	[25]
	Non-HDL Chol			[157]
	LDL Chol (LDL-C)			[157]
	Percentage of EPA and DHA in erythrocyte membrane		[164]	
Indices of weight control and body composition	BMI	[78,156,163,165]		[21,157]
	Obesity	[25,162,166,167,168,169]		
	Body fat	[163]		
	Anorexia of aging	[172]		
	Insufficient dietary intake and trace metals levels in the elderly	[173,174,175,176]		
	Disturbed levels of appetite-relevant hormones	[102]		
	Increased food consumption	[102]		
	Higher intake of sugary beverages	[171]		
Meeting the criteria for metabolic syndrome	Metabolic burden	[156,166]		
	Metabolic syndrome	[25,26,162]		[195]
	Metabolic disorders	[163]		
Elements of cardiovascular function	Hypertension	[26,155]		[25]
	Systolic BP	[21,69,78,157,177]		[158]
	Diastolic BP	[21,88]		[78,157,158]
	Heart rate (HR)		[21]	
	Cardiac output (CO)		[21]	
	Total peripheral resistance (TPR)	[21]		
	Cardiac contractility		[21]	
	Autonomic cardiac control		[178]	
	Coronary heart disease (CHD)	[22]		
	Stroke	[22]		
	Cardiovascular disease	[153,163,179]		
	NYHA class	[182]		
	Cardiovascular mortality	[180,181]		
Markers of cognitive function	Dementia	[23]		
	Elevated cortical amyloid burden	[183]		
	BDNF		[184]	[196]
	Allopregnanolone			[184] (hypothesis)
	Structural Brain Correlates	[185]		
Elements of mental health	Depression	[26,88,182,186]		[78]
	Pain	[103]		
	Fatigue	[103]		
	Schizophrenia	[155]		
	Diagnosis of drug abuse/dependence	[155]		
	Number of drugs used	[155]		
	Trouble adjusting to hassles	[79]		
	Mental health outcome		[24,105,163]	
	Sleep quality		[21,46,88,105]	
	Health behavior		[166,177,188]	[21]
	Recreational drug use	[21]		
	Physical activity		[189]	
Genetic correlates of metabolic regulation	Genes involving anti-inflammatory GREs		[42,190]	
	Oxidative stress	[190]		
	Genes involving response elements for pro-inflammatory NF-κB/Rel transcription factors	[42]		
	Genes associated with DNA methylation and epigenetic changes	[191]		
	BDNF Val66Met polymorphism	[197]		
All-cause mortality		[24,27,28]		

Abbreviations. +: positive association, −: negative association, No: No relation, NS: non-significant relation, HPA: Hypothalamic-Pituitary-Adrenocortical, AUC_G_: Area Under The Curve with respect to Ground, DHEAs: Dehydroepiandrosterone Sulfate, T2DM: Type 2 Diabetes Mellitus, T1DM: Type 1 Diabetes Mellitus, HbA1c: Hemoglobin A1C, FBS: Fasting Blood Sugar, AKT: Protein Kinase B, Tg: Triglycerides, Chol: Cholesterol, HDL: High-Density Lipoprotein, LDL: Low-Density Lipoprotein, EPA: Eicosapentaenoic Acid, DHA: Docosahexaenoic Acid, BMI: Body mass index, BP: Blood Pressure, NYHA: New York Heart Association, BDNF: Brain-Derived Neurotrophic Factor, GRE: Glucocorticoid Response Element, NF-κB/Rel: Nuclear Factor Kappa-light-chain-enhancer of Activated B Cells/Rel Homology Domain, Val: Valine, Met: Methionine.

## Data Availability

Not applicable.
